# Annotated checklist of Albanian butterflies (Lepidoptera, Papilionoidea and Hesperioidea)

**DOI:** 10.3897/zookeys.323.5684

**Published:** 2013-08-13

**Authors:** Rudi Verovnik, Miloš Popović

**Affiliations:** 1 University of Ljubljana, Biotechnical Faculty, Department of Biology, Večna pot 111, 1000 Ljubljana, Slovenia; 2 HabiProt, Bulevar oslobođenja 106/34, 11040 Belgrade, Serbia

**Keywords:** Balkan Peninsula, review, biodiversity, taxonomy, conservation

## Abstract

The Republic of Albania has a rich diversity of flora and fauna. However, due to its political isolation, it has never been studied in great depth, and consequently, the existing list of butterfly species is outdated and in need of radical amendment. In addition to our personal data, we have studied the available literature, and can report a total of 196 butterfly species recorded from the country. For some of the species in the list we have given explanations for their inclusion and made other annotations. Doubtful records have been removed from the list, and changes in taxonomy have been updated and discussed separately. The purpose of our paper is to remove confusion and conflict regarding published records. However, the revised checklist should not be considered complete: it represents a starting point for further research.

## Introduction

The Republic of Albania is situated in the Balkan Peninsula, in south-eastern Europe. It is predominantly a mountainous country with a Mediterranean climate, lying entirely within the Mediterranean ‘hotspot’ zone of biodiversity (Cuttelod et al. 2008). Its fauna and flora is exceptionally rich (MMPAU 2007, Radford et al. 2011, Marka et al. 2012), undoubtedly due to its geographic position, the diversity of its landscape, its complex geology and altitudinal range (from sea level up to 2753 m on Korab peak), but the country has unquestionably been understudied. This may, in part, be attributed to its political isolation from the rest of Europe during the communist regime. In recent years, however, the situation has changed dramatically, and Albania has become more accessible to outsiders.

As in other European countries, the butterflies of Albania have been studied in more detail than other insect groups. However, there is a considerable disparity between the number of species that have been recorded. Many of these differences can be attributed to doubtful and undocumented records, along with changes in taxonomy and nomenclature. The number of species recorded from Albania varies from 167 (Rebel and Zerny 1931) to 180 (Misja and Kurrizi 1984). These differences are also demonstrated in the most recent lists of species compiled by Fauna Europaea (2012) and the Red Data book of European butterflies (Van Swaay and Warren 1999).

As a result of this confusion, and with the prospect of new surveys, we present a revised checklist of butterflies for Albania, compiled from available literature and personal records. An updated list is essential for further faunistic studies, and provides a foundation for butterfly conservation in Albania. We discuss the species we have excluded but mentioned in previous lists and provide, where necessary, annotations for some species we have included.

## A review of the published records

The first comprehensive overview of the butterfly fauna for Albania was compiled by Rebel and Zerny (1931), who listed 167 butterfly species for the country, providing personal records for most of the species. In some instances, most notably *Pontia chloridice* (Hübner, 1813), the list is based on material collected by other researchers or on previously published records. After World War II, research continued, including a German expedition in the 1960s. However, the published results from this survey included only butterflies from the family Hesperiidae (Alberti 1965) and the genus *Erebia* (Popescu-Gorj 1971), and added no new species to the list compiled by Rebel and Zerny (1931). Moucha (1963a, 1963b), who visited the country in 1959, also provided no new additions to the list.

The first local entomologist to publish a list of butterfly fauna was Murraj (1972), and he also compiled an identification key for the 93 listed species. Among these *Carterocephalus palaemon* (Pallas, 1771), *Heteropterus morpheus* (Pallas, 1771), *Muschampia tessellum* (Hübner, 1803), *Coenonympha glycerion* (Borkhausen, 1788), *Melanargia lachesis* (Hübner, 1790) and *Melitaea aurelia* Nickerl, 1850 were the first records for the country. They were not, however, included in any of the subsequent lists or faunistic reports. An updated list of butterfly species for Albania was collated by Misja and Kurrizi (1984), listing 180 species. Several new findings for the country were added, including *Danaus chrysippus* (Linnaeus, 1758) (published also as new for Albania by Luquet and Misja (1989)), *Euphydryas maturna* (Linnaeus, 1758), *Satyrus actaea* (Esper, 1781) and *Pseudochazara mamurra* (Herrich-Schäffer, 1852).

In recent years, further new species have been added by Beshkov (1995) and Abadijev and Beshkov (1996): *Muschampia proto* (Ochsenheimer, 1808), *Hipparchia senthes* (Fruhstorfer, 1908) and *Hipparchia volgensis* (Mazochin-Porshnjakov, 1952). During the authors’ survey in south-eastern Albania in July 2012, three more species were discovered: *Colias aurorina* Herrich-Schäffer, 1850, *Pieris balcana* Lorković, 1970 and *Apatura iris* (Linnaeus, 1758) (Verovnik and Popović 2013).

The butterfly fauna of Albania was summarized in the Red data book of European butterflies (Van Swaay and Warren 1999). Kastriot Misja from the Museum of Natural Sciences in Tirana provided the data for Albania. In the overview of distribution there were 173 butterfly species recorded in Albania. Among these, *Euchloe penia* (Freyer, 1852), *Nymphalis vaualbum* ([Denis & Schiffermüller], 1775), *Hipparchia hermione* (Linnaeus, 1764) and *Pseudochazara cingovskii* (Gross, 1973) were important new additions. However, as none of these records was published separately, their inclusion should be regarded as questionable. The credibility of the list is further questioned by several omissions of common species, the absence of *Vanessa cardui* (Linnaeus, 1758) and *Coenonympha pamphilus* (Linnaeus, 1758) being the most obvious examples. The Fauna Europaea (2012) list, which indicates 170 species as present in Albania, has several similar omissions, and a single, more plausible, addition of *Aricia artaxerxes* (Fabricius, 1793).

If we include all published and unconfirmed records, the total number of butterfly species for Albania amounts to 208. This provides a starting point for revision.

## The revised checklist of the butterflies of Albania

After a systematic revision of the butterflies recorded for Albania, and having included all recent taxonomic changes, the checklist contains a total of 196 butterflies. The nomenclature follows Van Swaay et al. (2010) and Fauna Europaea (2012). Species marked with an asterisk are discussed in annotations which follow the list.

### Family Hesperiidae

1. *Pyrgus malvae* (Linnaeus, 1758)

2. *Pyrgus alveus* (Hübner, [1803])*

3. *Pyrgus armoricanus* (Oberthür, 1910)

4. *Pyrgus serratulae* (Rambur, 1839)

5. *Pyrgus cinarae* (Rambur, 1839)

6. *Pyrgus sidae* (Esper, 1784)

7. *Pyrgus carthami* (Hübner, [1813])

8. *Spialia orbifer* (Hübner, [1823])*

9. *Spialia phlomidis* (Herrich-Schäffer, [1845])

10. *Muschampia proto* (Ochsenheimer, 1808)

11. *Muschampia tessellum* (Hübner, [1803])*

12. *Carcharodus alceae* (Esper, [1780])

13. *Carcharodus lavatherae* (Esper, [1783])

14. *Carcharodus floccifera* (Zeller, 1847)

15. *Carcharodus orientalis* Reverdin, 1913

16. *Erynnis tages* (Linnaeus, 1758)

17. *Erynnis marloyi* (Boisduval, [1834])

18. *Carterocephalus palaemon* (Pallas, 1771)*

19. *Heteropterus morpheus* (Pallas, 1771)*

20. *Thymelicus acteon* (Rottemburg, 1775)

21. *Thymelicus lineola* (Ochsenheimer, 1808)

22. *Thymelicus sylvestris* (Poda, 1761)

23. *Hesperia comma* (Linnaeus, 1758)

24. *Ochlodes sylvanus* (Esper, 1777)

25. *Gegenes nostrodamus* (Fabricius, 1793)

26. *Gegenes pumilio* (Hoffmannsegg, 1804)

### Family Papilionidae

27. *Papilio machaon* Linnaeus, 1758

28. *Papilio alexanor* Esper, 1800

29. *Iphiclides podalirius* (Linnaeus, 1758)

30. *Zerynthia cerisy* (Godart, 1824)

31. *Zerynthia polyxena* ([Denis & Schiffermüller], 1775)

32. *Parnassius apollo* (Linnaeus, 1758)

33. *Parnassius mnemosyne* (Linnaeus, 1758)

### Family Pieridae

34. *Aporia crataegi* (Linnaeus, 1758)

35. *Pieris brassicae* (Linnaeus, 1758)

36. *Pieris rapae* (Linnaeus, 1758)

37. *Pieris mannii* (Mayer, 1851)

38. *Pieris ergane* (Geyer, [1828])

39. *Pieris balcana* Lorković, 1970

40. *Pieris napi* (Linnaeus, 1758)

41. *Pieris krueperi* Staudinger, 1860

42. *Pontia edusa* (Fabricius, 1777)*

43. *Pontia chloridice* (Hübner, [1813])

44. *Euchloe ausonia* (Hübner, [1804])

45. *Anthocharis cardamines* (Linnaeus, 1758)

46. *Anthocharis gruneri* Herrich-Schäffer, [1851]

47. *Colias aurorina* Herrich-Schäffer, 1850

48. *Colias hyale* (Linnaeus, 1758)*

49. *Colias alfacariensis* Ribbe, 1905

50. *Colias croceus* (Fourcroy, 1785)

51. *Gonepteryx rhamni* (Linnaeus, 1758)

52. *Gonepteryx cleopatra* (Linnaeus, 1767)

53. *Gonepteryx farinosa* (Zeller, 1847)

54. *Leptidea sinapis* (Linnaeus, 1758)

55. *Leptidea duponcheli* (Staudinger, 1871)

### Family Riodinidae

56. *Hamearis lucina* (Linnaeus, 1758)

### Family Lycaenidae

57. *Thecla betulae* (Linnaeus, 1758)

58. *Favonius quercus* (Linnaeus, 1758)

59. *Satyrium acaciae* (Fabricius, 1787)

60. *Satyrium ilicis* (Esper, 1779)

61. *Satyrium spini* ([Denis & Schiffermüller], 1775)

62. *Satyrium w-album* (Knoch, 1782)

63. *Satyrium pruni* (Linnaeus, 1758)

64. *Callophrys rubi* (Linnaeus, 1758)

65. *Lycaena phlaeas* (Linnaeus, 1761)

66. *Lycaena dispar* (Haworth, 1802)

67. *Lycaena virgaureae* (Linnaeus, 1758)

68. *Lycaena ottomanus* (Lefèbvre, 1830)

69. *Lycaena tityrus* (Poda, 1761)

70. *Lycaena alciphron* (Rottemburg, 1775)

71. *Lycaena thersamon* (Esper, 1784)

72. *Lycaena candens* (Herrich-Schäffer, 1844)*

73. *Lampides boeticus* (Linnaeus, 1767)

74. *Leptotes pirithous* (Linnaeus, 1767)

75. *Tarucus balkanica* (Freyer, 1844)

76. *Cupido argiades* (Pallas, 1771)

77. *Cupido decolorata* (Staudinger, 1886)

78. *Cupido minimus* (Fuessly, 1775)

79. *Cupido osiris* (Meigen, 1829)

80. *Celastrina argiolus* (Linnaeus, 1758)

81. *Glaucopsyche alexis* (Poda, 1761)

82. *Phengaris alcon* ([Denis & Schiffermüller], 1775)

83. *Phengaris arion* (Linnaeus, 1758)

84. *Iolana iolas* (Ochsenheimer, 1816)

85. *Scolitantides orion* (Pallas, 1771)

86. *Pseudophilotes vicrama* (Moore, 1865)

87. *Plebejus sephirus* (Frivaldzky, 1835)

88. *Plebejus argyrognomon* (Bergsträsser, 1779)

89. *Plebejus argus* (Linnaeus, 1758)

90. *Plebejus idas* (Linnaeus, 1761)

91. *Aricia eumedon* (Esper, [1780])

92. *Aricia agestis* ([Denis & Schiffermüller], 1775)

93. *Aricia artaxerxes* (Fabricius, 1793)*

94. *Aricia anteros* (Freyer, 1838)

95. *Cyaniris semiargus* (Rottemburg, 1775)

96. *Polyommatus damon* ([Denis & Schiffermüller], 1775)

97. *Polyommatus ripartii* (Freyer, 1830)

98. *Polyommatus admetus* (Esper, [1783])

99. *Polyommatus escheri* (Hübner, [1823])

100. *Polyommatus amandus* (Schneider, 1792)

101. *Polyommatus thersites* (Cantener, 1835)

102. *Polyommatus dorylas* ([Denis & Schiffermüller], 1775)

103. *Polyommatus daphnis* ([Denis & Schiffermüller], 1775)

104. *Polyommatus coridon* (Poda, 1761)

105. *Polyommatus bellargus* (Rottemburg, 1775)

106. *Polyommatus icarus* (Rottemburg, 1775)

107. *Polyommatus eros* (Ochsenheimer, 1808)*

### Family Nymphalidae

108. *Libythea celtis* (Laicharting, 1782)

109. *Danaus chrysippus* (Linnaeus, 1758)

110. *Charaxes jasius* (Linnaeus, 1767)

111. *Apatura iris* (Linnaeus, 1758)

112. *Apatura ilia* ([Denis & Schiffermüller], 1775)

113. *Limenitis reducta* Staudinger, 1901

114. *Neptis rivularis* (Scopoli, 1763)

115. *Nymphalis antiopa* (Linnaeus, 1758)

116. *Nymphalis polychloros* (Linnaeus, 1758)

117. *Nymphalis xanthomelas* (Esper, 1781)

118. *Aglais io* (Linnaeus, 1758)

119. *Aglais urticae* (Linnaeus, 1758)

120. *Vanessa atalanta* (Linnaeus, 1758)

121. *Vanessa cardui* (Linnaeus, 1758)

122. *Issoria lathonia* (Linnaeus, 1758)

123. *Polygonia c-album* (Linnaeus, 1758)

124. *Polygonia egea* (Cramer, 1775)

125. *Argynnis pandora* ([Denis & Schiffermüller], 1775)

126. *Argynnis paphia* (Linnaeus, 1758)

127. *Argynnis aglaja* (Linnaeus, 1758)

128. *Argynnis adippe* ([Denis & Schiffermüller], 1775)

129. *Argynnis niobe* (Linnaeus, 1758)

130. *Brenthis hecate* ([Denis & Schiffermüller], 1775)

131. *Brenthis daphne* (Bergsträsser, 1780)

132. *Brenthis ino* (Rottemburg, 1775)

133. *Boloria pales* ([Denis & Schiffermüller], 1775)

134. *Boloria graeca* (Staudinger, 1870)

135. *Boloria titania* (Esper, [1793])

136. *Boloria euphrosyne* (Linnaeus, 1758)

137. *Boloria dia* (Linnaeus, 1767)

138. *Melitaea cinxia* (Linnaeus, 1758)

139. *Melitaea phoebe* ([Denis & Schiffermüller], 1775)

140. *Melitaea didyma* (Esper, 1779)

141. *Melitaea trivia* ([Denis & Schiffermüller], 1775)

142. *Melitaea athalia* (Rottemburg, 1775)

143. *Melitaea aurelia* Nickerl, 1850*

144. *Euphydryas maturna* (Linnaeus, 1758)

145. *Euphydryas aurinia* (Rottemburg, 1775)

146. *Melanargia galathea* (Linnaeus, 1758)

147. *Melanargia russiae* (Esper, [1783])

148. *Melanargia larissa* (Geyer, [1828])

149. *Hipparchia syriaca* (Staudinger, 1871)*

150. *Hipparchia fagi* (Scopoli, 1763)

151. *Hipparchia volgensis* (Mazochin-Porshnjakov, 1952)

152. *Hipparchia semele* (Linnaeus, 1758)*

153. *Hipparchia senthes* (Fruhstorfer, 1908)

154. *Hipparchia fatua* Freyer, 1844

155. *Hipparchia statilinus* (Hufnagel, 1766)

156. *Chazara briseis* (Linnaeus, 1764)

157. *Pseudochazara geyeri* (Herrich-Schäffer, [1846])

158. *Pseudochazara anthelea* (Hübner, [1824])

159. *Pseudochazara mniszechii* (Herrich-Schäffer, [1851])*

160. *Pseudochazara amymone* Brown, 1976*

161. *Satyrus ferula* (Fabricius, 1793)

162. *Minois dryas* (Scopoli, 1763)

163. *Brintesia circe* (Fabricius, 1775)

164. *Arethusana arethusa* ([Denis & Schiffermüller], 1775)

165. *Erebia ligea* (Linnaeus, 1758)

166. *Erebia euryale* (Esper, [1805])

167. *Erebia epiphron* (Knoch, 1783)

168. *Erebia aethiops* (Esper, 1777)

169. *Erebia triarius* (de Prunner, 1798)

170. *Erebia medusa* ([Denis & Schiffermüller], 1775)

171. *Erebia gorge* (Hübner, [1804])

172. *Erebia rhodopensis* Nicholl, 1900

173. *Erebia cassioides* (Reiner & Hochenwarth, 1792)*

174. *Erebia ottomana* Herrich-Schäffer, [1847]

175. *Erebia pronoe* (Esper, [1780])

176. *Erebia melas* (Herbst, 1796)

177. *Erebia oeme* (Hübner, [1804])

178. *Erebia pandrose* (Borkhausen, 1788)

179. *Maniola jurtina* (Linnaeus, 1758)

180. *Hyponephele lycaon* (Rottemburg, 1775)

181. *Hyponephele lupinus* (Costa, 1836)

182. *Aphantopus hyperantus* (Linnaeus, 1758)

183. *Pyronia tithonus* (Linnaeus, 1767)

184. *Pyronia cecilia* (Vallantin, 1894)

185. *Coenonympha rhodopensis* Elwes, 1900*

186. *Coenonympha pamphilus* (Linnaeus, 1758)

187. *Coenonympha arcania* (Linnaeus, 1761)

188. *Coenonympha orientalis* Rebel, 1910

189. *Coenonympha leander* (Esper, 1784)

190. *Coenonympha glycerion* (Borkhausen, 1788)

191. *Pararge aegeria* (Linnaeus, 1758)

192. *Lasiommata megera* (Linnaeus, 1767)

193. *Lasiommata petropolitana* (Fabricius, 1787)

194. *Lasiommata maera* (Linnaeus, 1758)

195. *Kirinia roxelana* (Cramer, 1777)

196. *Kirinia climene* (Esper, [1783])

### Annotations to the list

The status of the following species is clarified. They are numbered according to their order in the checklist:

2. *Pyrgus alveus*

Rebel and Zerny (1931) listed *Pyrgus bellieri* Oberthür, 1910 for Albania. This is a western Mediterranean species whose presence in the Balkan Peninsula is highly doubtful. Alberti (1965) noted that the record is confirmed by dissection of the genitalia, thus it would be important to check its presence at Mt. Beshtriq (Mt. Pashtrik). At present most of the Pasthrik mountain is situated in Kosovo, not in Albania.

8. *Spialia orbifer*

*Spialia sertorius* (Hoffmannsegg, 1804) was listed for Albania by Misja and Kurrizi (1984). The Red Data book of European butterflies (Van Swaay and Warren 1999) lists both *Spialia sertorius* and *Spialia orbifer* for Albania. The south-eastern limit of *Spialia sertorius* in Europe is the northern Adriatic coast and Krk island in Croatia (Jakšić 1988, Habeler 2003), thus its presence in Albania is highly unlikely.

11. *Muschampia tessellum*

The species is listed for Albania only by Murraj (1972), and confirmation of this record is needed. It is known to occur in neighbouring northern Greece (Pamperis 2009) and from the Republic of Macedonia (Schaider and Jakšić 1989); therefore, its presence in the south-eastern part of Albania is possible.

18. *Carterocephalus palaemon*

The species is listed for Albania only by Murraj (1972) and requires confirmation. It is known from southern Serbia (Popović and Đurić 2011) and was recently discovered in Macedonia (Verovnik and Micevski 2008), thus its presence in the mountains of northern Albania is plausible.

19. *Heteropterus morpheus*

The species is listed for Albania only by Murraj (1972), and confirmation of the record is required. It is known to occur in the northern part of Montenegro (Sijarić et al. 1984) and in all probability is present in the mountainous parts of north-west Albania.

42. *Pontia edusa*

Its sister species *Pontia daplidice* (Linnaeus, 1758) is mentioned in several recent and historical lists (Rebel and Zerny 1931, Murraj 1972, Misja and Kurrizi 1984). The separate species status of *Pontia edusa*, which occurs in Eastern Europe, was not widely accepted until recently. *Pontia daplidice* is now credited as only flying in western part of Europe, not reaching the Balkan Peninsula. However, recent records from Cyprus indicate the possibility of a much wider distribution of *Pontia daplidice* in Europe (John et al. 2013).

48. *Colias hyale*

Records from this far South in the Balkan Peninsula are doubtful, and possibly refer to *Colias alfacariensis*. Nevertheless, the species does occur in central Serbia (Popović and Đurić 2011), and due to its migratory habit could potentially reach the northern part of Albania.

72. *Lycaena candens*

This species is listed for Albania only in Fauna Europaea (2012). However, the historical records for *Lycaena hippothoe* made by earlier authors (Rebel and Zerny 1931, Murraj 1972, Misja and Kurrizi 1984) should, in all probability, be referred to as *Lycaena candens*. It is highly unlikely that *Lycaena hippothoe* is found in Albania, as the closest confirmed records are from northern Bosnia (Lorković and Mihljević 1988) and north-western Serbia (Popović and Đurić 2011).

93. *Aricia artaxerxes*

Apart from its inclusion in Fauna Europaea (2012), the presence of this species in Albania has not appeared in any published record. During our surveys, we found the species on the north-western slopes of Mt. Grammos in 2012, confirming its presence in Albania.

107. *Polyommatus eros*

Only the subspecies *Polyommatus eros eroides* (Frivaldszky, 1835) is known to occur in Albania. The nominate subspecies could potentially be found in the calcareous high mountains on the border with Montenegro, where it is known from the Durmitor Mts. (Sijarić et al. 1984). Based on molecular studies, *Polyommatus eroides* has recently been downgraded to subspecies rank, due to a lack of genetic differentiation from *Polyommatus eros* (Vodolazhsky and Stradomsky 2008, Wiemers et al. 2010).

143. *Melitaea aurelia*

The species is mentioned for Albania only by Murraj (1972), and confirmation of the record is required. It has recently been found in the Republic of Macedonia (Micevski et al. 2009), also near the Albanian border on Mt. Galičica (Krpač et al. 2011). Its presence in Albania is very probable.

149. *Hipparchia syriaca*

Although mentioned as a separate species by Rebel and Zerny (1931), the taxon was incorrectly listed as *Hipparchia alcyone* (Murraj 1972, Misja and Kurrizi 1984) or *Hipparchia hermione* in subsequent lists (Van Swaay and Warren 1999). Gaskin (1990) and Beshkov (1995) correctly identified *Hipparchia syriaca* (Staudinger, 1871) as the species from this taxon group present in Albania.

152. *Hipparchia semele*

The exact distribution of this species in the southern and eastern part of the Balkan Peninsula is unknown, and the presence of two additional morphologically, almost indistinguishable, species, *Hipparchia volgensis* and *Hipparchia senthes*,makes identification difficult. Both these species have been recorded for Albania (Beshkov 1995, Abadijev and Beshkov 1996). The presence of *Hipparchia semele* in the mountains of the north-western part of the country is likely, and needs to be checked by future surveys.

159. *Pseudochazara amymone*

Although its status as a species is questionable, we follow the decision taken by Van Swaay et al. (2010), to treat it as a separate species. Its presence in southern Albania was recently reported by Eckweiler (2012). However, it was first reported from Albania as *Pseudochazara mamurra* by Misja and Kurrizi (1984).

160. *Pseudochazara mniszechii*

Based on external morphology and genitalia, the taxon *Pseudochazara tisiphone* (Brown, 1980) from northern Greece and Albania is considered to be conspecific with *Pseudochazara mniszechii* (Hesselbarth et al. 1995). It was originally described by Brown (1980) as a subspecies of *Pseudochazara cingovskii* (Gross, 1973) from Greece, and is listed as such for Albania by Van Swaay and Warren (1999). The first detailed records for Albania are mentioned in Tshikolovets (2011) from the environs of Korçë and Kolonjë.

173. *Erebia cassioides*

*Erebia cassioides* is included for Albania only in the list of Red Data book of European butterflies (Van Swaay and Warren 1999). However, some of the records for *Erebia tyndarus* (Esper, 1781) – in particular those of the f. *macedonica*, recorded by Rebel and Zerny (1931) – are actually records of *Erebia cassioides*. Two subspecies of *Erebia cassioides* are listed for Albania in the literature: *illyrica* Lorković, 1953 and *illyromacedonica* Lorković, 1953. *Erebia cassioides illyrica* is known from Bosnia and Hercegovina, Montenegro and the northern Albanian Alps, while *Erebia cassioides illyromacedonica* is mentioned for Macedonia, from the mountains on the border with Albania: Shar, Korab and Jakupica Mts. (Sijarić et al. 1984).

185. *Coenonympha rhodopensis*

*Coenonympha tullia* (Muller, 1764) is listed for Albania by early authors (Rebel and Zerny 1931, Murraj 1972, Misja and Kurrizi 1984), who did not consider *Coenonympha rhodopensis* as a separate species. *Coenonympha rhodopensis* was also observed during our survey on Mt. Grammos in 2012.

## Discussion

In comparison with countries in other parts of Europe, the butterfly fauna of Albania is very rich. Greece, which has been more intensively studied over the years, hosts 235 species of butterflies (Pamperis 2009) and has the most diverse butterfly fauna in the Balkan Peninsula. After recent surveys, however, the Republic of Macedonia, with 203 species (Verovnik and Micevski 2008, Micevski et al. 2009, Verovnik et al. 2010) and Serbia, with 198 species (Dinca et al. 2010, Popović and Đurić 2010, Popović and Milenković 2012, Popović et al. in press, Popović unpublished data), are not far behind. Montenegro, which in terms of landmass is much smaller, is the least studied of Albania’s neighbouring countries, with around 160 recorded species (Sijarić 1984).

There are several additional species listed for Albania in the available literature that have not been included in the current checklist: *Melanargia lachesis* (Hübner, 1790), listed by Murraj (1972), and *Satyrus actaea* (Esper, 1781), recorded by Misja and Kurrizi (1984), are both exclusively western European species; therefore, their presence in Albania is highly unlikely. Two species, *Euchloe penia* (Freyer, 1852) and *Nymphalis vaualbum* ([Denis & Schiffermüller], 1775), are listed only in the Red Data book of European butterflies (Van Swaay and Warren 1999). Both of them are remarkable species, and their occurrence in Albania, in all probability, would have been published separately. However, we can find no reference to them in the available literature. Therefore, they are currently not listed for Albania, but their presence cannot be entirely ruled out.

Certainly, the list of Albanian butterflies is far from complete. There are several species that are known to be present just across the border in neighbouring countries, and are likely to occur in Albania. *Apatura metis* Freyer, 1829 is one obvious example. It is known from north-western Greece (Mairiaux and Hutsebaut 1997) and Skadar Lake in Montenegro (Jakšić 1988) near the border with Albania. Another interesting species that is expected to be found in Albania is *Cacyreus marshalli* (Butler, 1898), which has been recently been reported from the Croatian coast (Kosmač and Verovnik 2009) and Greece (Anastassiu et al. 2010). We hope that our contribution will initiate more interest in the diverse butterfly fauna of Albania and trigger further butterfly studies in this country.
